# A randomised controlled trial of an open lung strategy with staircase recruitment, titrated PEEP and targeted low airway pressures in patients with acute respiratory distress syndrome

**DOI:** 10.1186/cc10249

**Published:** 2011-06-02

**Authors:** Carol L Hodgson, David V Tuxen, Andrew R Davies, Michael J Bailey, Alisa M Higgins, Anne E Holland, Jenny L Keating, David V Pilcher, Andrew J Westbrook, David J Cooper, Alistair D Nichol

**Affiliations:** 1 Intensive Care Unit, The Alfred, Commercial Rd, Melbourne, VIC 3181, Australia; 2 Australian and New Zealand Intensive Care Research Centre, Department of Epidemiology and Preventative Medicine, Monash University, Commercial Rd, Melbourne, VIC 3004, Australia; 3 School of Physiotherapy, La Trobe University, Commercial Rd, Melbourne, VIC 3004, Australia; 4 School of Primary Health Care, Faculty of Medicine, Nursing and Health Sciences, Monash University, Frankston, VIC 3199, Australia; 5 Physiotherapy Department, The Alfred, Commercial Rd, Melbourne, VIC 3181, Australia

## Abstract

**Introduction:**

Tidal volume and plateau pressure minimisation are the standard components of a protective lung ventilation strategy for patients with acute respiratory distress syndrome (ARDS). Open lung strategies, including higher positive end-expiratory pressure (PEEP) and recruitment manoeuvres to date have not proven efficacious. This study examines the effectiveness and safety of a novel open lung strategy, which includes permissive hypercapnia, staircase recruitment manoeuvres (SRM) and low airway pressure with PEEP titration.

**Method:**

Twenty ARDS patients were randomised to treatment or ARDSnet control ventilation strategies. The treatment group received SRM with decremental PEEP titration and targeted plateau pressure < 30 cm H_2_O. Gas exchange and lung compliance were measured daily for 7 days and plasma cytokines in the first 24 hours and on days 1, 3, 5 and 7 (mean ± SE). Duration of ventilation, ICU stay and hospital stay (median and interquartile range) and hospital survival were determined.

**Results:**

There were significant overall differences between groups when considering plasma IL-8 and TNF-α. For plasma IL-8, the control group was 41% higher than the treatment group over the seven-day period (ratio 1.41 (1.11 to 1.79), *P *= 0.01), while for TNF-α the control group was 20% higher over the seven-day period (ratio 1.20 (1.01 to 1.42) *P *= 0.05). PaO_2_/F_I_O_2 _(204 ± 9 versus 165 ± 9 mmHg, *P *= 0.005) and static lung compliance (49.1 ± 2.9 versus 33.7 ± 2.7 mls/cm H_2_O, *P *< 0.001) were higher in the treatment group than the control group over seven days. There was no difference in duration of ventilation (180 (87 to 298) versus 341 (131 to 351) hrs, *P *= 0.13), duration of ICU stay (9.9 (5.6 to 14.8) versus 16.0 (8.1 to 19.3) days, *P *= 0.19) and duration of hospital stay (17.9 (13.7 to 34.5) versus 24.7 (20.5 to 39.8) days, *P *= 0.16) between the treatment and control groups.

**Conclusions:**

This open lung strategy was associated with greater amelioration in some systemic cytokines, improved oxygenation and lung compliance over seven days. A larger trial powered to examine clinically-meaningful outcomes is warranted.

**Trial registration:**

ACTRN12607000465459

## Introduction

Acute respiratory distress syndrome (ARDS) is an inflammatory condition of the lungs that is associated with high mortality [1]. Mechanical ventilation is a life supporting intervention that aims to maintain gas exchange in these patients, but it can also augment or initiate lung injury [2]. Lung-protective mechanical ventilation strategies that aim to minimise tidal volume and plateau pressure have been the predominant intervention associated with improved patient survival [3,4].

Clinicians frequently use high positive end-expiratory pressure (PEEP) to improve alveolar recruitment in patients with ARDS. PEEP aims to counter the pulmonary shunt due to increased lung collapse resulting from inflammation. High PEEP maintains functional residual capacity and improves oxygenation [5,6] and may even have an effect on reducing mortality associated with ARDS [7,8]. The best strategy to set optimal PEEP for an individual patient has not yet been established [9,10].

It is unclear whether lung recruitment manoeuvres (LRM) add benefit to low tidal volume protective ventilation strategies in ARDS [11,12]. The most commonly used LRM requires the application of sustained continuous positive airway pressure (CPAP) at 35 to 40 cm H_2_O for 40 seconds [2,13,14]. However, this LRM method can be uncomfortable, may induce circulatory depression and has not been associated with improved outcomes in patients with ARDS [13-15].

We previously demonstrated that a staircase recruitment manoeuvre (SRM) was safe and effective in improving oxygenation and lung compliance for up to one hour in patients with ARDS[16]. The SRM involves a progressive increase in PEEP (up to 40 cm H_2_O) over several minutes with mandatory pressure control ventilation, resulting in intermittent higher pressures (55 cm H_2_O) for longer duration and increased alveolar recruitment compared with static recruitment methods [16]. Borges and co-workers found that oxygenation benefits of the SRM can be maintained for up to six hours with the application of "optimal" PEEP using a PEEP titration manoeuvre [17]. To our knowledge the effect of an open lung strategy, which includes SRM and PEEP titration, on inflammatory markers or physiological indices has not been investigated beyond six hours in patients with ARDS.

The potentially deleterious higher airway pressures observed in previous strategies that incorporated high PEEP and LRMs may be avoided by reducing tidal volume, a practice that may require permissive hypercapnia. It has been demonstrated in animals and humans that the acidosis induced by this hypercapnia, independent of any changes in ventilator strategy, may also confer benefit in ARDS [18-20].

The aim of this pilot trial was to compare an open lung pressure control ventilation strategy that utilised SRM, high PEEP and permissive hypercapnia to limit airway pressures (PHARLAP; Permissive Hypercapnia, Alveolar Recruitment, Low Airway Pressures) with a control strategy (conventional ARDSnet 'protective' volume controlled ventilation [21]) in patients with ARDS to determine the effect on inflammatory cytokines, physiological lung injury (arterial oxygenation and lung compliance) and rates of barotrauma over a seven-day period.

## Materials and methods

This pilot randomised, controlled, parallel-group study was conducted between January 2008 and October 2009. The Human Research Ethics Committees of The Alfred Hospital and Monash University approved the study protocol. Informed consent was obtained from each patient's next of kin.

Twenty mechanically ventilated patients with ARDS [22] in the Intensive Care Unit of the Alfred Hospital were enrolled. Patients were randomised to treatment (PHARLAP) or control groups using sequentially numbered sealed opaque envelopes, generated by computerised random block schedule. Patients were stratified by the diagnosis of severe sepsis according to ACCP/SCCM Consensus Conference guidelines [23-25].

Inclusion criteria were the diagnosis of ARDS [26,27], age > 15 years, and the presence of both an intra-arterial line and central venous catheter. Patients were excluded if they had chest trauma, an intercostal catheter with air leak, a pneumothorax on chest x-ray, bronchospasm on auscultation, raised intracranial pressure, mean arterial pressure ≤60 mmHg, significant arrhythmias or were ventilated for longer than 72 hours.

### Interventions

#### PHARLAP ventilation strategy

The PHARLAP strategy included pressure control ventilation (PCV), with plateau pressures < 30 cm H_2_O while delivering tidal volumes of less than 6 mls/kg ideal body weight (IBW) with patients in a supine position with 30 degrees head of bed elevation. The fraction of inspired oxygen (F_I_O_2_) was adjusted until the continuously monitored oxygen saturation was 90 to 92%. For the SRM, the high pressure was set to 15 cm H_2_O above the PEEP, which was increased in a stepwise manner to 20, then 30 and then 40 cm H_2_O every two minutes, and then reduced to 25, then 22.5, then 20, then 17.5 or then an absolute minimum of 15 cm H_2_O every three minutes until a decrease in SaO_2 _≥ 1% from maximum SaO_2 _was observed. This was defined as the derecruitment point. PEEP was then increased to 40 cm H_2_O for one minute and returned to a PEEP level 2.5 cm H_2_O above the derecruitment point (which was then defined as optimal PEEP). Stepwise increases in PEEP did not continue if the patient became bradycardic or tachycardic (< 60 or > 140 beats per minute), developed a new arrhythmia, became hypotensive (systolic blood pressure < 80 mmHg) or became hypoxaemic (SaO_2 _< 85%). Following this SRM the tidal volume was adjusted to achieve a tidal volume ≤ 6 mls/kg IBW and a plateau pressure ≤ 30 cm H_2_O. Hypercapnia was tolerated and acidosis was only treated if the pH was less than 7.15 by increasing respiratory rate to a maximum of 38 breaths per minute. The PHARLAP group received one SRM daily (with decremental PEEP titration) until the patient was deemed ready for weaning. In addition, PEEP was transiently elevated to 40 cm H_2_O (with PCV at 15 cm H_2_O) for one minute if oxygen desaturation ≤ 90% occurred or after disconnection from the ventilator.

Patients were assessed daily for weaning readiness. Weaning was commenced in both groups when all of the following occurred: respiratory rate < 35 breaths per minute, PaO_2 _> 60 mm Hg, SpO_2 _> 90% with fraction of inspired oxygen < 0.4 and PEEP < 10 cm H_2_O, mean arterial pressure > 60 mm Hg without inotrope infusions or sedatives.

#### Control ventilation strategy

The control group was treated using the ARDSnet protocol, with assist control ventilation and FiO_2_/PEEP titration [21]. Tidal volumes were limited to 6 mls/kg, plateau pressures < 30 cm H_2_O. Acidosis (pH < 7.3) was actively managed by increasing minute ventilation. PCV was not used, and recruitment manoeuvres were only allowed if the patient met the criteria for use of a rescue therapy, which was when the patient was receiving FiO_2 _≥ 0.9, and the treating clinicians considered one necessary.

### Outcome measures

Plasma interleukin-6 (IL-6), interleukin-1β (IL-1β), interleukin-8 (IL-8) and TNF-α concentrations were measured from arterial blood samples taken at baseline, three hours after randomisation and then on days 1, 3, 5 and 7. Samples were immediately centrifuged at 1,500 g for 10 minutes and plasma aspirated and stored at -70°C. Cytokines were detected using commercially available enzyme-linked immune-absorbent assays (ELISA, R&D Systems, Inc., Minneapolis, MN, USA).

Peak and plateau pressure, tidal volume, respiratory rate, PEEP, heart rate and rhythm, central venous pressure, blood pressure, inotrope dosage and arterial blood gases were measured at baseline, 1, 3, 6 and 24 hours and then daily during the period of mechanical ventilation up to seven days. Derived variables were PaO_2_/F_I_O_2 _ratio and static lung compliance. Length of stay (in ICU and in hospital), length of mechanical ventilation and hospital survival for both groups were recorded.

### Sample size

The sample size was one of convenience as this was a pilot study. We estimated that 10 patients per group would provide > 80% power to detect a difference of one standard deviation in cytokine levels, with a two-sided test for differences, a *P-*value of 0.01 whilst assuming an Intraclass Correlation of 0.2 between baseline level and Day 3. The intention to treat principle was utilised.

### Statistical analysis

All outcomes were initially assessed for normality and log-transformed where appropriate. Group comparisons were made using chi-square tests for equal proportion, Student *t*-tests for normally distributed data and Wilcoxon rank sum tests otherwise, with normally distributed data reported as means ± standard errors and non-normal data reported as medians (interquartile range). Group comparisons over time were performed using repeated measures analysis of variance fitting an overall group effect, a time effect and a group by time interaction to ascertain if the groups behaved differently over time. As cytokine measurements were found to be well approximated by log-normal distributions, results have been graphed as geometric means (95% CI) with differences reported as ratios (95% CI). Where baseline differences were found to exist, results were analysed using analysis of covariance with baseline values as a covariate. All models were fitted using the PROC Mixed procedure in SAS (SAS Version 9.1 SAS Institute Inc., Cary, NC, USA). To account for possible bias arising from differing extubation or dropout rates between groups, additional sensitivity analyses were conducted for compliance and oxygenation with patients carrying their last observation forward. A two-sided *P*-value ≤0.05 was considered statistically significant.

## Results

**Figure 1:**
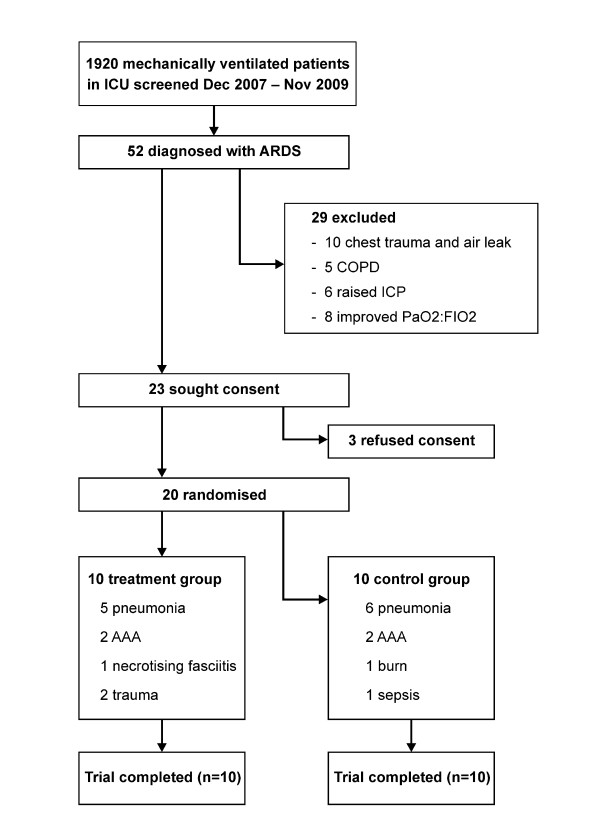
**Patient flow diagram**. AAA, abdominal aortic aneurysm; COPD, chronic obstructive lung disease; ICP, intracranial pressure; PaO_2_/F_I_O_2, _partial pressure of oxygen to inspired fraction of oxygen ratio.

Twenty patients with ARDS were enrolled (Figure 1). Baseline demographic data of the control and PHARLAP groups are displayed in Table 1. There were no statistically significant differences between the groups at baseline.

**Table 1:** Baseline demographic data (mean ± SE)

	PHARLAP	Control	*P*
Number in group	10	10	
Male, number	7	6	
Age, years	60 ± 5	58 ± 4	0.65
APACHE 2 score	20.1 ± 3	20.1 ± 2	0.99
APACHE 3 score	66.3 ± 8	64.8 ± 7	0.89
SOFA score	8.6 ± 0.9	8.4 ± 0.5	0.86
PaO_2_/F_I_O_2_, mmHg	155 ± 8	149 ± 12	0.65
Diagnostic group	5 pneumonia	6 pneumonia	
	2 AAA	2 AAA	
	1 necrotising fasciitis	1 burn	
	2 trauma	1 sepsis	
Static lung compliance, ml/cm H_2_O	45.8 ± 5.4	37.3 ± 5.4	0.48
PEEP, cm H_2_O	11.8 ± 0.7	14.2 ± 1.2	0.09

AAA, abdominal aortic aneurysm repair; APACHE, acute physiology and chronic health evaluation; PEEP, positive end expiratory pressure; SE, standard error, SOFA, sequential organ failure assessment score

In the PHARLAP group, all 10 patients received daily SRM with maximum PEEP of 40 cm H_2_O and a maximum plateau airway pressure of 55 cm H2O. Three patients transiently desaturated to < 90% at maximum PEEP of 40 cm H_2_O with no lasting adverse effects. There was no radiographic evidence of barotrauma during the seven day study period.

Two patients from the control group developed severe hypoxaemia (SaO_2 _≤ 90% whilst receiving FiO_2 _0.9 and PEEP 18) and received rescue therapies (a static recruitment manoeuvre in one and inhaled nitric oxide in the other). One patient in the control group died during the seven-day intervention period. Five patients in the PHARLAP group were weaned from mechanical ventilation within the seven days compared to three in the control group. At Day 7, there were five PHARLAP group patients and six control group patients who remained on mechanical ventilation.

**Figure 2:**
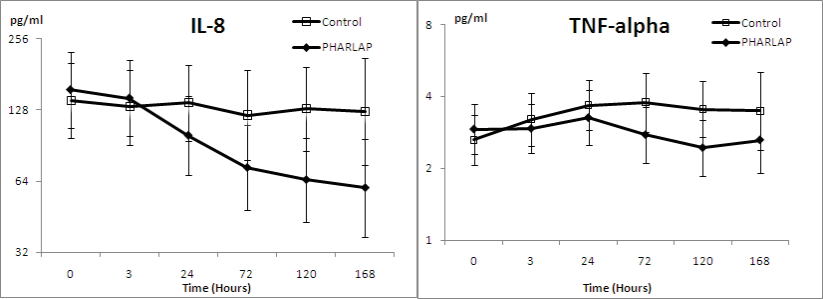
**IL-8 and TNF-α measured over 168 hours (seven days) reported as geometric means (95% CI)**. There was a significant overall difference in interleukin-8 and serum tumour necrosis factor-alpha between the treatment group and the control group over the seven-day period (*P *= 0.01 and *P *= 0.05 respectively).

There was evidence to suggest that some cytokine values differed between groups with plasma IL-8 and TNF-α levels being significantly lower in the PHARLAP group (Figure 2). Using an analysis of covariance with baseline values as a covariate, the overall levels of IL-8 over all time points were 41% higher in the control group compared to the PHARLAP group (ratio 1.41 (1.11 to 1.79), *P *= 0.01). Similarly, overall levels of TNF-α over all time points were 20% higher in the control group compared to the PHARLAP group (ratio 1.20 (1.01 to 1.42), *P *= 0.05). There were no statistically significant differences in IL-6 or IL-1β between the treatment and control groups.

**Figure 3:**
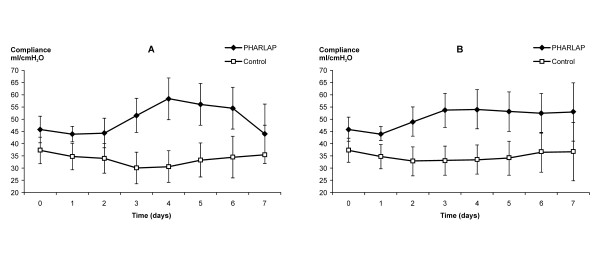
**Static lung compliance measured in ventilated patients for seven days (mean ± SE)**. There was a significant overall improvement in static lung compliance in the PHARLAP group compared to control group patients. **A **= missing data analysed as random *P *= 0.001, **B **= last observation carried forward *P *= 0.01.

**Figure 4:**
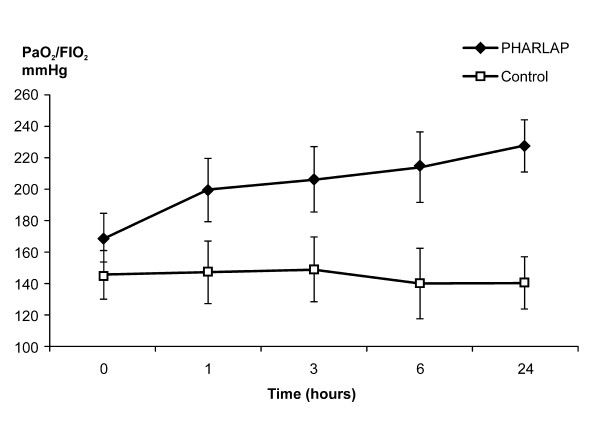
**PaO_2_/F_I_O_2 _measured over the first 24 hours in ventilated patients (mean ± SE)**. PHARLAP group had a significant overall increase in PaO_2_/F_I_O_2 _compared to control group patients (*P < 0.01).

**Figure 5:**
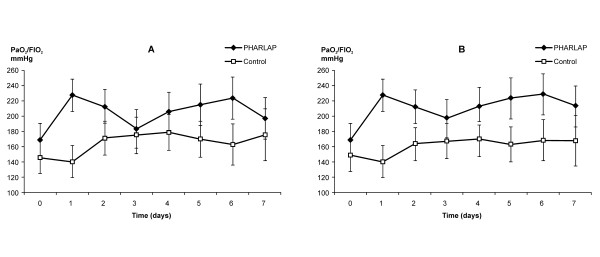
**PaO_2_/F_I_O_2 _measured over seven days in ventilated patients (mean ± SE)**. There was a significant overall improvement in PaO_2_/F_I_O_2 _ratio in PHARLAP compared to control group patients. **A **= missing data analysed as random, *P *= 0.005. **B **= last observation carried forward, *P *= 0.03.

There was a significant overall improvement in static lung compliance in the PHARLAP group compared to the control group over seven days (49.1 ± 2.9 versus 33.7 ± 2.7 mls/cm H_2_O, *P *< 0.001, Figure 3). PaO_2_/F_I_O_2 _was higher in the PHARLAP group than the control group over the first 24 hours (Figure 4) and over 7 days (204 ± 9 versus 165 ± 9 cm H_2_O, *P *= 0.005, Figure 5).

The PEEP was higher in the PHARLAP group over the first 24 hours (Table 2) and throughout the 7 days (PHARLAP 12 ± 0.5 cm H_2_O, control 9.5 ± 0.5, *P *= 0.004, Table 3) than the control group.

**Table 2:** Respiratory variables during the first 24 hrs of treatment (mean ± SE)

	1 hour		3 hours		6 hours		24 hours	
	PHARLAP	Control	PHARLAP	Control	PHARLAP	Control	PHARLAP	Control
V_T_, mls	519 ± 56	501 ± 30	517 ± 51	529 ± 58	529 ± 56	542 ± 49	463 ± 42	563 ± 65
RR, bpm	21 ± 2	21 ± 1	20 ± 2	21 ± 2	21 ± 2	21 ± 2	22 ± 2	20 ± 2
F_I_O_2_	0.47 ± 0.03	0.59 ± 0.04	0.5 ± 0.04	0.5 ± 0.03	0.4 ± 0.05	0.57 ± 0.05	0.4 ± 0.04	0.5 ± 0.04
PEEP, cm H_2_O	17.4 ± 1	11.6 ± 1	17.4 ± 1*	11 ± 0.5	16.7 ± 1*	10 ± 0.6	15 ± 1*	10 ± 0.5
Pplateau, cm H_2_O	28.9 ± 1.2	27.1 ± 1.2	28.3 ± 1.1	26.6 ± 1.1	29 ± 0.8	26 ± 0.8	27.6 ± 1.5	26.9 ± 1.4
Arterial pH	7.34 ± 0.02	7.36 ± 0.02	7.34 ± 0.02	7.34 ± 0.03	7.34 ± 0.02	7.36 ± 0.01	7.36 ± 0.01	7.35 ± 0.01
PaCO_2_, mm Hg	49 ± 5	46 ± 5	47 ± 4	48 ± 6	48 ± 3	45 ± 3	45 ± 3	46 ± 3

*P < 0.05 for differences between PHARLAP and control groups.

V_T_, tidal volume; RR, respiratory rate; F_I_O_2_, fraction of inspired oxygen; PEEP, positive end expiratory pressure; Pplateau, plateau pressure; PaO_2_/F_I_O_2_, partial pressure of oxygen to inspired fraction of oxygen ratio; PaCO_2_, partial pressure of carbon dioxide

**Table 3:** Respiratory variables during seven days of treatment (mean ± SE) * *P *< 0

	Baseline		Day 1		Day 3		Day 7	
	**PHARLAP**	**Control**	**PHARLAP**	**Control**	**PHARLAP**	**Control**	**PHARLAP**	**Control**

**V_T_, mls**	519 ± 56	501 ± 30	463 ± 42	563 ± 65	586 ± 58	511 ± 55	528 ± 76	579 ± 78
**RR, bpm**	21 ± 2	21 ± 1	22 ± 2	20 ± 2	19 ± 2	24 ± 2	22 ± 2	22 ± 2
**FiO_2_**	0.48 ± 0.06	0.57 ± 0.06	0.4 ± 0.04	0.5 ± 0.04	0.5 ± 0.07	0.55 ± 0.06	0.5 ± 0.07	0.5 ± 0.09
**PEEP**, **cm H_2_O**	11.8 ± 0.7	14.2 ± 1.2	15 ± 1*	10 ± 0.5	12.1 ± 1.5	9.3 ± 1.4	8.5 ± 1.8	7.8 ± 2.0
**Pplateau, cmH_2_O**	28.4 ± 1.5	29 ± 1.5	27.6 ± 1.5	26.9 ± 1.4	24.2 ± 2.4	24 ± 2.1	21 ± 2.9	20. ± 3.4
**Arterial pH**	7.34 ± 0.02	7.36 ± 0.02	7.36 ± 0.01	7.35 ± 0.01	7.38 ± 0.03	7.44 ± 0.03	7.42 ± 0.03	7.42 ± 0.04
**PaCO_2_, mmHg**	49 ± 5	46 ± 5	45 ± 3	46 ± 3	47.6 ± 3.7	44 ± 3.5	43.3 ± 4	56.5 ± 5

Vt, tidal volume; RR, respiratory rate; F_I_O_2_, fraction of inspired oxygen; PEEP, positive end expiratory pressure; Pplateau, plateau pressure; PaCO_2_, partial pressure of carbon dioxide

There were no other significant differences between the groups (Table 3) in respiratory and haemodynamic variables, peak or plateau pressures, pH, PaCO_2 _or SOFA scores during the seven-day period. Of note, the mean plateau pressures were less than 30 cm H_2_O throughout the study in both groups and the plateau pressures were no higher in the PHARLAP group than the control group.

There were no differences in length of ventilation, length of stay in ICU and length of stay in hospital, or hospital survival (Table 4).

**Table 4:** Outcomes

	PHARLAP	Control	*P*
**Hospital mortality, number**	3	2	0.61
**LOV, hours**	180 (87 to 298)	341 (131 to 351)	0.13
**ICU LOS, days**	9.9 (5.6 to 14.8)	16.0 (8.1 to 19.3)	0.19
**Hospital LOS, days**	17.9 (13.7 to 34.5)	24.7 (20.5 to 39.8)	0.16
**Barotrauma, number**	0	0	
**Rescue therapies, number of patients**	0	2	0.46
**SOFA score (Day 7)**	8.6 ± 0.3	8.4 ± 0.6	0.27

LOV, length of ventilation; ICU LOS, intensive care length of stay; LOS, length of stay; SOFA, sequential organ failure assessment

## Discussion

This pilot, randomised controlled study examined the efficacy and safety of a multi-faceted open lung mechanical ventilation strategy that included permissive hypercapnia, staircase recruitment manoeuvres, decremental PEEP titration, low airway pressure and pressure control ventilation in patients with ARDS. The strategy appeared safe and was associated with ameliorations in plasma IL-8 and TNF-α levels, improved static lung compliance and improved oxygenation over a seven-day period. Although some cytokines were not significantly ameliorated (IL-6 and IL-1β) and unsurprisingly given the size of the study there were no significant differences in duration of mechanical ventilation, ICU stay and hospital stay.

Static lung compliance decreased by nearly 30% in the control strategy group over the first 24 hours and remained low for the duration of the study compared with the PHARLAP strategy, which actually resulted in an increase in compliance. These suggest a greater degree of lung recruitment was sustained throughout the study period in the PHARLAP group, an effect which may be important in ARDS to minimise the potential negative effects of ventilator induced lung injury.

Systemic arterial oxygenation, as measured by the PaO_2_/F_I_O_2, _was improved by the PHARLAP strategy and maintained for seven days. The beneficial effects of PEEP on oxygenation have been demonstrated by systematic review and include an association with improved survival in patients with ARDS [8]. It is unclear from our results whether the improved oxygenation was as a result of the increased PEEP, the SRM, both, or another aspect of our multi-pronged strategy. However, the results of this trial expand on the previous work by our group which demonstrated that the SRM with decremental optimal PEEP titration improved lung compliance and oxygenation over a one hour period in patients with ARDS [16].

The shorter duration of mechanical ventilation in the PHARLAP group resulted in smaller group size contributing to the mean values of PaO_2_/F_I_O_2 _and compliance as days progressed. This may have given the incorrect appearance of decreasing differences between the two groups especially considering that patients with better PaO_2_/F_I_O_2 _and compliance values are more likely to be extubated. We have attempted to correct for this by including a sensitivity analysis with last observation carried forward (Figures 3 and 5). In both analyses the differences between the PHARLAP and the control ventilation groups were statistically significant with the PHARLAP group having higher PaO_2_/F_I_O_2 _and static lung compliance over seven days.

It is as yet unclear if these physiological improvements would translate into clinically meaningful outcomes such as improved survival. However, in our study the use of rescue therapies for severe hypoxaemia was only required in the control group. Two of the patients in the control group required nitric oxide and the application PEEP levels higher than specified by the control group strategy protocol to maintain adequate oxygenation.

Although the study protocol advocated permissive hypercapnia and low airway pressures as components of the PHARLAP strategy, the mean PaCO_2_, pH and plateau pressure values were similar in both the PHARLAP and control groups. This suggests that these factors were less likely to have been responsible for the different outcomes between the groups. The primary differences in strategies were the application of the recruitment manoeuvre and the higher PEEP level with a lower driving pressure (a consequence of higher PEEP and unchanged plateau pressure) in the PHARLAP group. This is in contrast to several randomised trials [13,14,28], in all of which the treatment groups had a higher plateau pressure in association with a higher PEEP level, an important factor which may have confounded that ability of these high PEEP (± LRM) studies to detect a difference between groups. Importantly, our strategy achieved similar peak and plateau airway pressures in both groups despite increased levels of PEEP in the PHARLAP group.

Transient desaturation at maximum PEEP during SRMs with subsequent augmentation of oxygen saturation higher than baseline with PEEP reduction has previously been described by our group [16] and by others [17]. In this study, maximum PEEP was associated with transient desaturation in 3 of the 10 patients who received SRMs. There were no other adverse events reported. Transient desaturation does not indicate a failure of the lungs to respond to a recruitment manoeuvre [16]. The PHARLAP strategy improved lung compliance and oxygenation despite transient desaturation in these three patients.

Lung recruitment manoeuvres that involve high airway pressures to achieve and maintain lung recruitment have the potential to cause over-distension [29]. Plasma levels of IL-6, IL-1β, IL-8 and TNF-α were analysed to determine if the SRM caused an increase in inflammatory markers, which might reflect the systemic effects of over-distension lung injury. Our results showed that the PHARLAP strategy resulted in an overall reduction of plasma IL-8 and TNF-α over seven days that may have indicated a protective benefit associated with the treatment strategy. To ensure that observed differences between groups for IL-8 and TNF-α were not due to baseline imbalances, an analysis of covariance was conducted with baseline values used as covariates. There were no significant differences for IL-6 and IL-1β, which may reflect the large heterogeneity of the patient population, the small sample size, or that some cytokine levels are not affected by this mechanical ventilation strategy.

Although this study was not adequately powered to determine clinically-meaningful outcomes, it is interesting to note that the PHARLAP strategy was associated with what might be considered trends (*P *< 0.20) towards shorter duration of mechanical ventilation, ICU stay and hospital stay. We feel such pilot study results warrant investigation in a larger randomized trial. If the PHARLAP strategy is effective, it may be a safe and cost effective treatment strategy for patients with ARDS.

This study has a number of limitations. The unblinded nature of the study, coupled with the use of adjunctive interventions at the discretion of the intensive care physician in the case of severe hypoxaemia, may have confounded our results. There are several possible mechanisms for a decrease in SaO_2 _observed during the SRM other than atelectasis and increased shunt, which may lead to a false assumption of developing airway closure. It is possible that during the incremental pressure of the SRM there were reduced tidal volumes resulting in increased PaCO_2 _and arterial desaturation; however, in a previous study by our group the PaCO_2 _had returned to baseline levels at the point of determination of optimal PEEP [16]. We performed this study in a single-centre, which facilitated rigorous education and consistent implementation of the strategy, but may decrease the generalisability of the results to other populations. The small sample size meant the study was underpowered to determine differences in length of mechanical ventilation, ICU stay and hospital stay. It also meant that despite random allocation the static lung compliance at baseline was slightly higher and the PEEP was slightly lower in the PHARLAP group (Table 1). These differences were not statistically significant, but may have influenced our results.

## Conclusions

This randomized controlled trial showed that a multi-faceted open lung strategy that was based on staircase recruitment manoeuvres and decremental PEEP titration improved plasma cytokines (IL-8 and TNF-α), static lung compliance and oxygenation over seven days. There were no differences in duration of mechanical ventilation, ICU stay or hospital stay; however, further investigation in a larger randomized trial is warranted.

## Key messages

• An open lung strategy including staircase recruitment and PEEP titration improved plasma cytokines, static lung compliance and oxygenation over seven days

• An open lung strategy including staircase recruitment and PEEP titration was safe

• Open lung ventilation was associated with a trend for reduced duration of ventilation that requires investigation in a larger trial

• Open lung ventilation was associated with less use of rescue therapies

## Abbreviations

ARDS: acute respiratory distress syndrome; ARDSnet: Acute Respiratory Distress Syndrome Network; CI: confidence interval; F_I_O_2_: fraction of inspired oxygen; IBW: ideal body weight; ICU: intensive care unit; IL-6: interleukin-6; IL-8: interleukin-8; IL-1β: interleukin-1β; PaCO_2_: partial pressure of carbon dioxide; PaO_2_: partial pressure of oxygen; PaO_2_/F_I_O_2_: ratio of partial pressure of oxygen to fraction of inspired oxygen; PCV: pressure control ventilation; PEEP: positive end expiratory pressure; PHARLAP: open lung ventilation strategy with Permissive Hypercapnia and Alveolar Recruitment and Low Airway Pressure; SaO_2_: arterial oxygen saturation; SE: standard error; SRM: staircase recruitment manoeuvre; SOFA: Sequential Organ Failure Assessment score; TNF-α: tumour necrosis factor-alpha; VCV: volume controlled ventilation.

## Competing interests

The authors declare that they have no competing interests.

## Authors' contributions

CH, AN and DT conceived and participated in the design of the study, collected the data, and drafted the manuscript. JC and AD conceived of the study, participated in its design and coordination, and manuscript preparation. AW and DP collected data and participated in the manuscript preparation. MB participated in the manuscript preparation and data analysis. JK and AEH and AMH participated in study design, and manuscript preparation. CH and AN coordinated the study. All authors read and approved the final manuscript.
